# Midpalatal Suture Maturation in Relation to Age, Sex, and Facial Skeletal Growth Patterns: A CBCT Study

**DOI:** 10.3390/children11081013

**Published:** 2024-08-20

**Authors:** Felice Festa, Mario Festa, Silvia Medori, Giada Perrella, Pasquale Valentini, Giorgio Bolino, Monica Macrì

**Affiliations:** 1 Department of Innovative Technologies in Medicine & Dentistry, University “G. d’Annunzio” of Chieti-Pescara, 66100 Chieti, Italy; 2 Department of Anatomic, Histologic, Medical-Legal Sciences and of Locomotor System, Rome University Sapienza, 00185 Rome, Italy; 3 Department of Economics, University “G. d’Annunzio” of Chieti-Pescara, 66100 Chieti, Italy

**Keywords:** 3D, midpalatal suture, maxillary expansion, CBCT

## Abstract

**Simple Summary:**

The treatment of a transverse maxillary constriction is a challenge for orthodontists, as the expansion approach depends on the midpalatal suture maturation. A conventional palatal expander provides skeletal effects in an unfused suture, while surgically or miniscrew-assisted expansions are suggested in a closed suture. The midpalatal suture development is affected by multiple variables, such as age, sex, and facial growth patterns. Consequently, we conducted a CBCT study among 263 patients aged from 8 to 20 in order to evaluate the relation between midpalatal suture maturation and age, sex, and vertical and sagittal growth patterns. The midpalatal suture was classified into five stages from A to E according to the progressively increasing maturation stage. The youngest individuals, the male participants, and the hyperdivergent subjects tended to show lower stages. Therefore, our study provided a further analysis of the potential predictors involved in midpalatal suture maturation that are useful to the clinician to choose the most effective expansion approach.

**Abstract:**

Background. The evaluation of midpalatal suture maturation is essential to undertake the most predictable maxillary expansion approach. Several factors, such as age, gender, and facial growth patterns, seem to be involved in midpalatal suture staging and, consequently, in its opening; however, the link between these variables and the stages of midpalatal suture development remains poorly understood. Our study aimed to analyse the midpalatal suture maturation in relation to age, sex, and skeletal growth patterns by CBCT. Methods. We enrolled 263 patients (119 males and 144 females) aged from 8 to 20 years. The midpalatal suture maturation was defined according to Angelieri et al.’s classification using a low-dose CBCT. The chi-square test and linear regression were applied to investigate the suture stages by age, sex, and vertical and sagittal growth patterns. Results. Stage A was present in 8- and 9-year-olds with a larger prevalence in boys, while the prevalence of stage E increased progressively with age. Stage D was the most prevalent in our sample. The statistical analysis described that stage A was more likely in the youngest subjects, and stage E in the oldest participants. The males tended to have lower maturation stages. Moreover, the hypodivergent and normodivergent subjects tended to have higher maturation stages, while Class III was more likely in subjects in stages D or E. Conclusions. A total of 127 patients were in stages A, B, and C, showing an unfused suture. In young individuals, the opening of the midpalatal suture leads to a proper facial growth development by correcting the transverse superior hypoplasia. The midpalatal sutural maturation classification was related to age, sex, and divergence.

## 1. Introduction

A transverse maxillary deficiency is a frequent skeletal anomaly compromising facial growth and indicates a poorly developed maxilla due to genetic, environmental, and/or functional factors [1,2]. The diagnosis of transverse skeletal deficit is generally based on clinical and radiological assessments [3]. 

The rapid palatal expansion is the main procedure that aims to correct a transverse superior hypoplasia and, more generally, a reduced development of the facial middle third. The opening of the midpalatal suture in early ages has a positive impact on residual growth, avoiding later, more complex therapies. The first palatal expansion was described by Angell in 1860 [4]. After a century, Haas proposed the current rapid palatal expander, namely a tooth-borne device capable of opening the midpalatal suture by means of the lengthening of collagenous fibres and, subsequently, the apposition of bone [5]. This appliance requires orthopaedic forces since the maxilla is connected to the cranium through multiple sutures, such as the fronto-maxillary, zygomatic-maxillary, zygomatic-frontal, and zygomatic-temporal sutures. In fact, it is not only the midpalatal suture, but also the circummaxillary sutures, offering the main resistances and limitations opposing the palatal disjunction [6]. The efficacy of the rapid palatal expansion is affected by the midpalatal maturation stages. The most reliable results can be noticed in growing patients, in whom a complete suture ossification has not yet occurred. As a consequence of the suture fusion progression, the resistance of the suture to opening increases with age. In fact, the adults generally show dental rather than skeletal effects following a palatal expansion treatment; therefore, a surgically assisted rapid palatal expansion (SARPE) or miniscrew-assisted rapid palatal expansion (MARPE) is often suggested in post-pubertal patients [7,8].

The individual skeletal maturation can be detected through a hand-wrist radiography or an evaluation of cervical vertebrae in lateral radiography [9,10]. Nevertheless, these biological indicators allow for defining the mandibular growth more correctly than the midpalatal suture maturation, although some authors have noticed a correlation between cervical vertebral maturation and palatal suture, mostly in patients of prepubertal ages [11,12]. 

Different approaches have been proposed to state midpalatal suture maturation. Histological studies can provide precise information; however, their routine application is difficult as an invasive biopsy is required [13]. Among the radiological methods, frontal or occlusal radiographies, computed tomography, and cone beam computed tomography (CBCT) have been used in previous research [14,15]. Conventional frontal and occlusal radiographs may present the overlap of contiguous anatomies, while computed tomography offers precise images with high radiation doses. To overcome these shortcomings, CBCT provides a 3D visualisation of orofacial structures minimising the patient’s exposure to ionising radiation [16]. Based on previous histological research, in 2013, Angelieri et al. classified the palatal suture, evaluated in the axial plane, into five stages (from A to E) according to the sutural morphology by means of CBCT scans [17]. Conventional rapid palatal disjunction can be applied in patients in the A, B, and C stages, although, in the C stage, lower skeletal effects could be observed. A surgical procedure represents a better treatment approach in the D and E stages, where the suture fusion has occurred [18]. Previous works recommended conventional expansion before puberty; on the other hand, some authors suggested the rapid maxillary expander approach also in adults [19,20].

The chronological age, the individuals’ ethnicities, the nutritional deficiencies, and the sagittal and vertical skeletal facial growth patterns as well as the circummaxillary structures are involved in modelling palatal suture ossification. Most studies have dealt with the relationship between midpalatal suture maturation and age. Some authors found no link, while other researchers highlighted a direct correlation [21,22]. Moreover, Tonello et al. reported no statical differences concerning sex in midpalatal suture development [23]. Few papers analysed the effect of skeletal growth pattern on midpalatal suture maturation and focused mainly on the vertical proportion; however, discordant outcomes were obtained. Hence, the potential effects of these predictors on palatal maturation staging are currently not fully clear. 

Therefore, the purpose of the present study was to determine the ossification level of the midpalatal suture in children, adolescents, and young adults from an Italian sample by CBCT scans according to the method described by Angelieri et al. [17]. We also investigated whether a relationship between palatal suture development and age or gender could be possible. Lastly, we aimed to analyse the midpalatal suture maturation in relation to the patient’s sagittal and vertical facial growth patterns.

## 2. Materials and Methods

CBCT scans were obtained from 263 patients (119 males and 144 females) aged between 8 and 20 years, with an average of 14 years and 2 months (Table 1).

Ethical approval (number 23, 8 November 2018) was obtained by the Independent Ethics Committee of Chieti hospital. The study protocol was drawn following the European Union Good Practice Rules and the Helsinki Declaration.

The sample was selected according to inclusion/exclusion criteria from patients who needed an orthodontic therapy in the Department of Innovative Technologies in Medicine & Dentistry at “G. d’Annunzio” University of Chieti-Pescara from October 2020 to December 2023. We estimated the size of our sample by the formula to assess a proportion, i.e., the possibility to detect an unfused midpalatal suture in subjects older than 18 years, with the confidence level of 95%, precision of 5%, and 10% proportion of this eventuality (data from a precedent pilot test). A total of 138 CBCT scans represented the minimum sample needed to conduct our analysis. Subjects with malocclusion of any sagittal and vertical growth patterns and good quality CBCT images were included. Patients with facial asymmetries, cleft lip and palate, systemic diseases or trauma, syndromic anomalies, previous orthodontic/orthopaedic therapies, or blurred CBCT images were excluded. All subjects or the legally authorized representative provided the written informed consent. The justification for a low-dose CBCT was the presence of retained teeth, transverse maxillary deficiency, skeletal malocclusion, or evaluation of root position and radicular resorptions. None of our participants were recruited purely for the current study aim.

CBCT scans were acquired by Planmeca Promax^®^ 3D MID unit (Planmeca Oy, Helsinki, Finland) with a low dose protocol: exposure time of 15 s, 80 kVp, 5 mA, 35 microSievert (μSv), field of view (FOV) of 240 × 190 mm, and normal image resolution [24]. During the CBCT scan, the patient was seated with the back perpendicular to the ground and the head fixed through the ear rods in the external auditory meatus, gazing into a mirror positioned 1.5 m in front of him in order to position the head in the Natural Head Position (NHP). The NHP is a repeatable and specified posture in the morphological analysis [25]. Every participant was instructed to remain motionless and to maintain centric occlusion with a light lip contact during the radiographic exam. Then, DICOM files were analysed and interpreted through Dolphin Imaging 3D software 11.9 (Dolphin Imaging & Management Solutions, Chatsworth, CA, USA). In order to achieve a consistent and repeatable 3D analysis, it is essential to predefine the patient’s head position. The NHP was used to orientate the skull image in the three planes of space perpendicular to each other, as described in our previous studies: “the transverse plane coincides with the Frankfurt plane (FH), a plane passing through two points: Orbital (Or) and Porion (Po); the sagittal plane coincides with the mid-sagittal plane (MSP), a plane perpendicular to the FH plane and passing through two points: Crista Galli (Cg) and Basion (Ba); the coronal plane coincides with the anteroposterior (PO) plane, perpendicular to the FH and MSP, passing through the right and left portion” [26].

Finally, we obtained the virtual 2D radiograms. In the present study, the superior submento-vertex and lateral teleradiography were considered to assess the midpalatal suture maturation and skeletal growth patterns, respectively.

**Figure 1:**
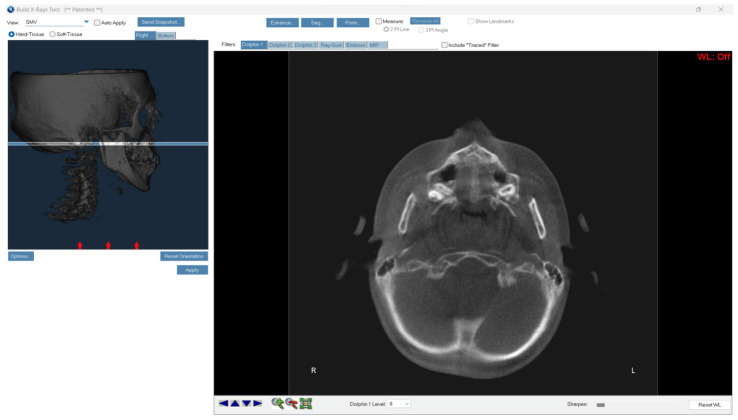
Superior submento-vertex obtained through the widget present in Dolphin Imaging 3D software. The most central submento-vertex delimited between the nasal and oral surfaces was selected.

Regarding the superior submento-vertex, in the sagittal plane, the midsagittal cross-sectional slice was used to position the palate horizontally, parallel to the software’s horizontal red line, as described by Angelieri et al. [17]. The most central cross-sectional slice delimited between the nasal and oral surfaces was obtained through the widget present in Dolphin Imaging 3D software (Figure 1). Two axial slices were used in subjects with a curved or thicker palate, since a single slice could not offer a correct visualisation of all palatal portions: the anterior and posterior portions cannot be visualised in the same axial slice.

**Figure 2:**
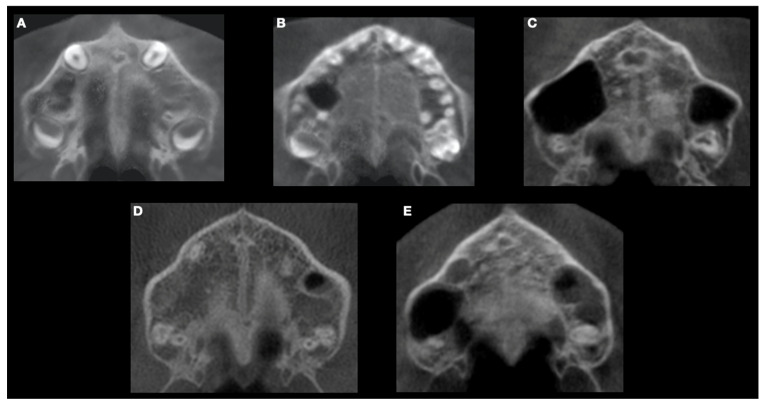
Classification of midpalatal suture maturation into five stages according to Angelieri et al. (**A**) The midpalatal suture appears as a relatively straight radiopaque line. (**B**) The midpalatal suture becomes a scalloped high-density line. (**C**) The midpalatal suture is seen as two radiopaque, scalloped and parallel lines, separated by small low-density areas. (**D**) The palatine bones are fused and the suture cannot be visualised in this region. (**E**) The fusion has also occurred anteriorly in the maxilla.

Each midpalatal suture was evaluated in the superior submento-vertex according to the five stages proposed by Angelieri et al.:

- Stage A refers to a relatively straight high-density line at the midline with no or little interdigitations;
- Stage B shows a scalloped high-density line at the midline with an irregular shape;
- Stage C presents two parallel, scalloped, high-density lines close to each other and separated in some areas by small low-density spaces;
- Stage D indicates a complete fusion in the palatine bone, where the midpalatal suture cannot be visualised;
- Stage E exhibits the midpalatal fusion also extended in the anterior portion of the palate, i.e., the complete palatal ossification has occurred (Figure 2) [17].

**Figure 3:**
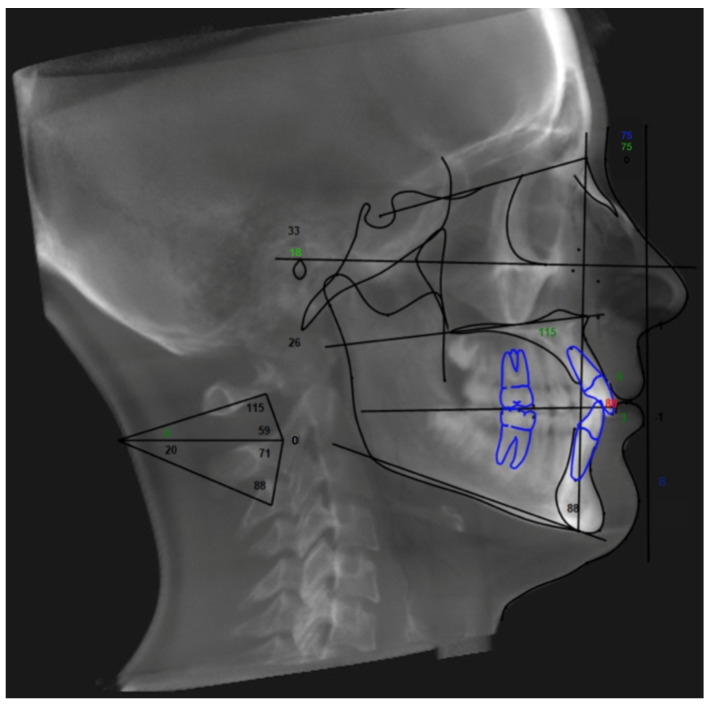
Cephalometric analysis according to McLaughlin, performed by means Dolphin Imaging 3D software. In the present study, we used SN-GoGn angle and ANB angle to determine the vertical proportion and sagittal growth pattern, respectively.

Concerning the skeletal growth patterns, the cephalometric analysis, according to McLaughlin, was performed on lateral teleradiography using Dolphin Imaging 3D software. After the insertion of the cephalometric points requested, the linear and angular values were automatically calculated (Figure 3).

For vertical skeletal growth pattern distinction, the participants were divided, according to the Sella-Nasion plane-mandibular plane angle, SN-GoGn angle, into hypodivergent (SN-GoGn angle < 27°), normodivergent (SN-GoGn angle = 33° ± 6°), and hyperdivergent (SN-GoGn angle > 39°) groups. For the sagittal growth pattern classification, the patients were differentiated, according to A point-Nasion-B point angle, ANB angle, into Class III (ANB angle < −0.4°), Class I (ANB angle = 2° ± 2.4°), and Class II (ANB angle > 4.4°).

Two blinded examiners, previously instructed to extract and interpret the axial and sagittal sections in Dolphin Imaging 3D software, defined midpalatal suture maturation and skeletal growth patterns in the same room under the identical light conditions by means of the same monitor between January and April 2024. The two observers had no information about participants’ age and gender; in fact, an experienced clinician had previously selected the patients according to the inclusion/exclusion criteria and assigned a number to each patient. These two blinded examiners were orthodontists who trained for a year to classify the midpalatal suture and to perform cephalometric analysis. During the training phase, the examiners had carefully studied the original work of Angelieri et al. and practiced on the cross sections. Any controversies were debated with the experienced clinician. 

For every participant, the age, sex, SN-GoGn angle, ANB angle, and midpalatal suture stage data were reported in an Excel spreadsheet (version 2019; Microsoft, Redmond, WA, USA).

The absolute (number) and relative (percentage) frequencies of midpalatal maturation stages and skeletal growth patterns were collected in tables.

The weighted kappa coefficient was calculated for the evaluation of inter-examiner measurement error.

The chi-square test and the linear regression analyses were used to analyse the suture stages by age, sex, and vertical and sagittal growth patterns. The level of significance was set at 5%. All statistical procedures were conducted with StataCorp. 2023. Stata Statistical Software: Release 18.

## 3. Results

The repeatability of the midpalatal suture maturation stage, SN-GoGn and ANB angles was evaluated using a weighted kappa. The respective values for these three parameters were 0.824, 0.876, and 0.853, demonstrating almost perfect agreement.

Linear regression analyses were conducted to estimate the associations between three independent variables—sex, midpalatal suture maturation stages, and facial skeletal growth patterns—and age, which was treated as a continuous outcome. For each regressor, we defined three reference categories: female, stage A, and hyperdivergent. Table 2 presents the results obtained from the linear regression models. The model demonstrated a satisfactory fit, with a coefficient of determination (R^2^) of 0.74 and a significant overall F-test (*p*-value < 0.000). The Cook’s distance for each observation was less than 1, indicating the absence of outliers in the dataset that could negatively impact the coefficient estimates.

The analysis demonstrates that the predictors of sex and midpalatal suture maturation have a significant impact on age, unlike midpalatal suture maturation, which does not appear to be a relevant predictor for the considered outcome. Specifically, it is observed that males are, on average, 0.94 years older than females.

Regarding midpalatal suture maturation, it is noted that as one progresses to a higher stage, there is an increase in age compared to the baseline (stage A). More specifically, individuals in stage E are expected to be 10.16 years older than those in stage A, while for stage D, the expected age is 7.21 years higher than it is for those classified as stage A. Similar considerations can be made for stages B and C. 

Table 3 shows the distribution of the midpalatal maturation stages by age and sex among all participants grouped by age.

We found stage C present until 15 and 19 years in the girls and the boys, respectively. On the other hand, stage D did not appear before the age of 11 in either sex, while stage E was observed three years earlier in the females than in the males. None of the five stages were detected in all age groups. Stage A showed the lowest prevalence (7.60%), while stage D was the most prevalent (34.98%) in our study sample.

In detail, stage A was present in 8- and 9-year-olds, with a larger prevalence in boys of both age groups. Stage B was observed until 13 years of age and was prevalent in 10-year-olds. Eleven girls and 14 boys showed stage B. Stage C was present in patients aged from 10 to 19 years. At age 10, only three females showed stage C, while a 19-year-old boy was in stage C.

Stage D was the most prevalent among all ages examined in our study, observed in 92 patients (51 females and 41 males). Similar gender prevalences were observed between 14 and 16 years of age. The highest prevalences were in patients aged from 15 to 17 years (15-year-olds, 64.70%; 16-year-olds, 61.54%; and 17-year-olds, 70.0%). Stage E was observed in 16.73% of patients. Its prevalence increased progressively with age: the lowest prevalence was detected at age 12 (2.44%) and the highest prevalence at age of 20 (61.9%).

**Figure 4:**
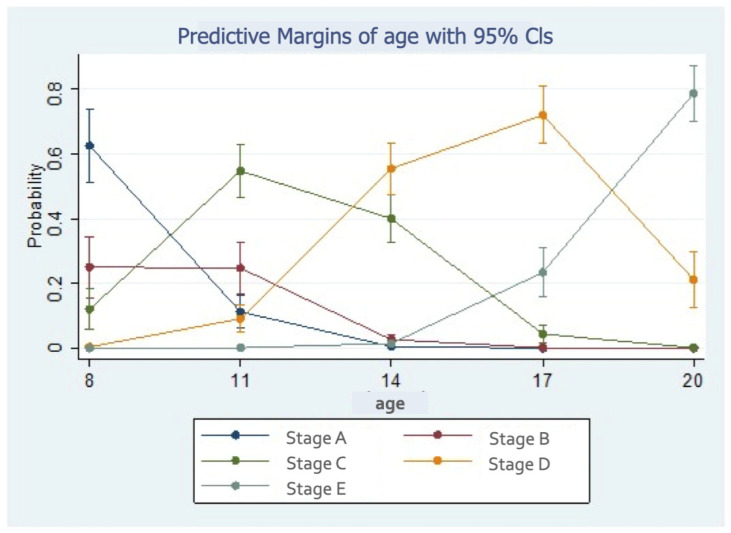
Probability of midpalatal maturation stages according to age.

Statistically, the probability of stage A tended to decrease with age; on the other hand, the probability of stage E tended to increase with age. The probability of stages C and D was increased in the youngest age groups and reduced in the oldest (Figure 4).

Evaluating the sex distribution, stages A, B, and C were most prevalent in males, while the latest stage was primarily detected in girls (Table 2). Stage C was present in 32.77% of males, while stage D was present in 34.45% of boys. The other stages showed similar prevalences (11.76% in stage B, 10.92 in stage A, and 10.10 in stage E). Females showed a higher prevalence in stage D (35.42%), followed by stage C (29.86%). The lowest prevalence (4.86%) was seen in stage A. The prevalence of stages E and B in girls were 22.22% and 7.64%, respectively. The girls showed stages related to a higher midpalatal suture maturation than the boys in each age group. 

**Figure 5:**
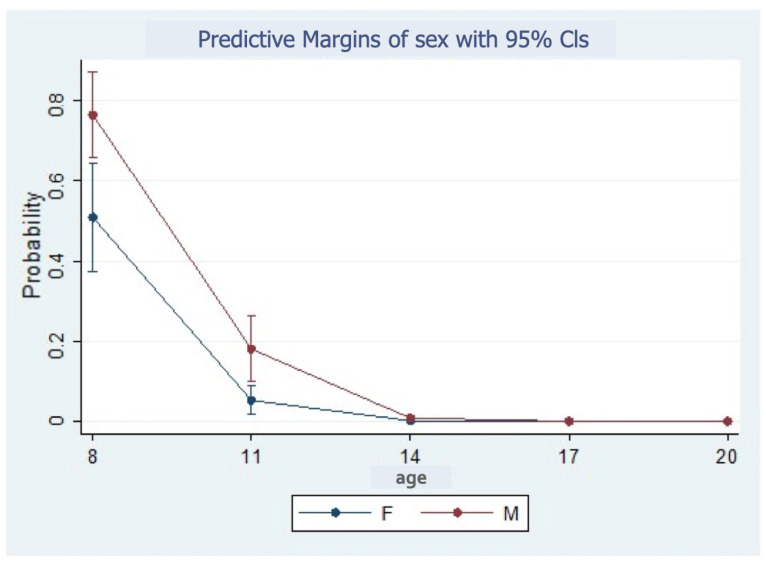
Probability of midpalatal maturation stage A according to sex.

The males tended to have lower maturation stages. The male individuals were more than twice as likely as the female individuals to say they were in stage A (10.47% versus 4.2%), about as likely to report they were in stage C (31.9% versus 30.7%), and about half as likely to say they were in stage D (12.8% versus 20.13%). Moreover, stage A was more likely in males than females, especially at ages 8 and 11 (Figure 5).

Concerning the vertical skeletal growth pattern, 45 patients were hypodivergent, 113 were normodivergent, and 105 were hyperdivergent, as summarised in Table 4. The lowest prevalence was observed in four hypodivergent boys at stages A and B (0.76%, respectively). The highest prevalence was observed in 47 hyperdivergent patients at stage C (17.87%).

**Figure 6:**
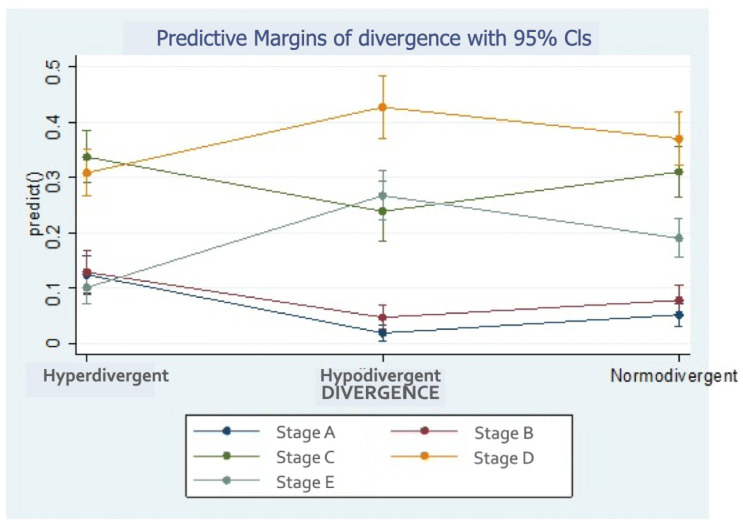
Probability of midpalatal maturation stages according to divergence.

The hypodivergent and normodivergent subjects tended to have higher maturation stages (Figure 6). In fact, the hyperdivergent individuals were more than six times as likely as the hypodivergent individuals to say they were stage A (12.38% versus 1.93%), and about half as likely to say they were in stage D (10.2% versus 26.7%).

Regarding the sagittal growth pattern, 86 patients had a skeletal Class I (32.7%), 136 patients had a skeletal Class II, and 41 patients had a skeletal Class III, as summarised in Table 5. The highest prevalence was observed in Class II at stage C (43 patients, 16. 35%) and D (40 patients, 15.21%). The lower prevalence was observed in Class III patients at stage A (five patients, 1.9%) and B (one patient, 0.38%). The patients presenting with skeletal Class I, II, and III showed a similar distribution in the different midpalatal maturation. There is a low percentage in stages A, B, and E and a higher concentration in stages C and D. The distribution of males and females across different skeletal classes in the various midpalatal maturation stages was almost equal, except in Class II in stage E, where the number of females (22 patients, 15.29%) was noticeably higher than that of males (four patients, 3.36%).

**Figure 7:**
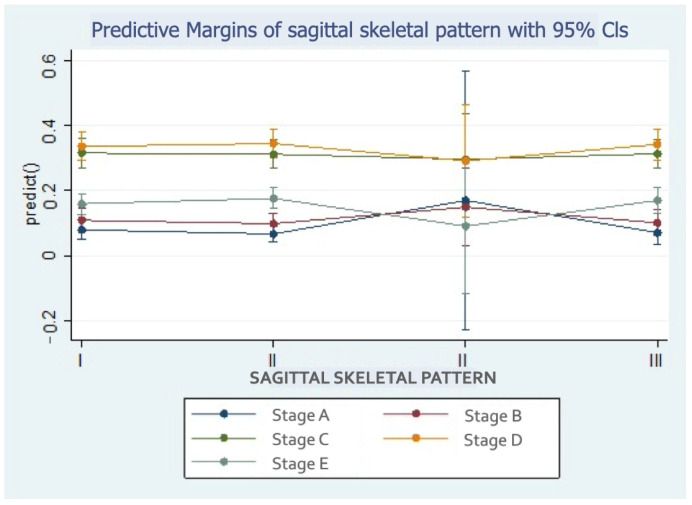
Probability of midpalatal maturation stages according to sagittal growth pattern.

Class III patients were more likely to be in stages D or E than in other stages (Figure 7).

To assess the predictive power of a model effectively, a commonly used method is cross-validation. This approach involves dividing the dataset into parts and then constructing a model using one portion while evaluating it on the other. The most prevalent technique for this is K-Fold cross-validation, which follows these steps:

(1) Randomly divide the sample into K equal parts.
(2) For the kth part, build the model using the remaining K − 1 parts of the data, and use this model to predict the outcome for each observation in the kth part.
(3) Repeat the previous step for k = 1, 2, …, K and combine the K sets of predictions to create a complete sample of actual and predicted values.
(4) Utilize the actual and predicted values to calculate any desired measures of goodness of fit.

In our specific case, we employ K = 10 for the cross-validation process. To evaluate the predictive performance, we use the testing set and consider two measures:

(1) The Root Mean Square Prediction Error (*RMSPE*), computed as the square root of the sum of squared differences between observed values (*Y_i_*) and predicted values (${\hat{Y}}_{i}$) for *N* observations.
  $$RMSPE=\sqrt{\sum_{i=1}^{N}{\left(Y_{i}-{\hat{Y}}_{i}\right)}^{2}}$$
(2) The Coefficient of Determination (*R*^2^), calculated as 1 minus the ratio of the sum of squared differences between observed values ($Y_{i}$) and predicted values (${\hat{Y}}_{i})$ to the sum of squared differences between observed values ($Y_{i}$) and their mean (${\bar{Y}}_{i}$).
  $$R^{2}=\;1-\sum_{i=1}^{N}{\left(Y_{i}-{\hat{Y}}_{i}\right)}^{2}/\sum_{i=1}^{N}{\left(Y_{i}-{\bar{Y}}_{i}\right)}^{2}$$

In our specific application, we obtained an *R*^2^ value of 0.70 and an *RMSPE* value of 1.89. These results indicate that the model exhibits a good predictive capability, and, on average, the model’s predictions have an error of 1.89 years. 

## 4. Discussion

The present study investigated whether a correlation between midpalatal suture maturation and age, gender, or skeletal growth patterns could be possible. Differently from other studies, ours encompassed a larger number of participants in a narrower age range. Our sample was homogenous: all patients have the same ethnicity and eating habits.

A transverse maxillary constriction compromises the maxillofacial complex growth, worsting the initial malocclusion. Therefore, the timely identification and proper treatment of an underdeveloped maxilla allow the recovery of a correct relationship between the skeletal bases, especially during early childhood [27,28]. Moreover, an early diagnosis avoids more complex and longer therapies in the later ages.

In patients with a constricted maxillary width, the rapid palatal expansion correcting the maxilla-mandibular discrepancy represents the most predictable approach in daily clinical practice; however, several factors could affect the final outcomes. Applying heavy forces in a palatal suture that is closing could lead to unwanted drawbacks, for instance, accentuated buccal tipping, gingival recession, root resorption, or fenestration of buccal cortex especially in posterior teeth [29]. Similarly, the SARPE protocol in subjects with an open suture could subject these patients to the unnecessary risks and costs associated with a more invasive procedure. It is worth pointing out that other therapeutic options, such as customised lingual brackets in both arches, have been recently investigated. Schmid et al. did not detect a significant buccolingual tipping in adult patients treated with a non-surgical approach, if compared with SARPE [30]. Therefore, it is essential to identify the midpalatal suture maturation in order to choose the most suitable treatment in relation to patient’s clinical features.

To date, the sutural classification proposed by Angelieri et al. still remains the most reliable approach to define midpalatal suture maturation as it is based on a complete evaluation of palatal morphology along its entire long axis through CBCT scan [17]. Indeed, CBCT is a useful radiological method for the clinician in the diagnostic phase as it allows to perform multiple slices on the axial plane at various levels and, hence, to precisely assess the suture maturation. 

Multiple variables, such as age, gender, and skeletal growth pattern, could affect the midpalatal suture maturation. In our study sample, all stages were detected, contrary to the findings of other authors who did not find stage A in the same age range of sample the (8–20 years). However, we did not notice all stages in a single age group. 

Regarding the age, in our study, we found a correlation between the maturation of the midpalatal suture and the chronological age. In fact, the probability of stage A tended to decrease with age, while the probability of stage E tended to increase with age.

Nevertheless, in the literature, no consensus regarding the age cut-off for a conventional expander protocol has yet been reached. Previous papers reported different midpalatal suture maturation stages in a single age group. Angelieri et al. found all five stages in individuals over the age of 11 and, in a later work, Angelieri et al. noticed an open suture in 12% of adults, demonstrating no statistic correlation between chronological age and sutural stages [31]. Therefore, the authors underlined the need for an individual sutural assessment by CBCT scans mainly in late adolescents in whom a palatal disjunction could have unreliable findings. Indeed, the skeletal effects can be achieved until 10–11 years, while in late puberal ages, the percentage of individuals in stage C increased and stages D and E were detected in females, reducing, thus, the skeletal effects of a conventional expander. Other later works supported the use of CBCT to investigate the palatal ossification, especially in young adults [32]. On the contrary, the chronological age was a relevant factor involved in midpalatal suture development in other papers [33]. Silva-Montero et al. underlined a correlation between age and suture maturation, although the authors found a small percentage of young adults with an unfused suture [22]. Chávez-Sevillano et al. noticed a positive correlation between midpalatal ossification and age, especially in males [34].

In our analysis, the females tend to mature earlier than the males in the same age group, which is in accordance with previous studies. For example, stage E appeared at the age of 12 in the girls and 15 in the boys. Indeed, skeletal maturity occurs more prematurely in females than in males, albeit with both genders showing analogous bone density until middle age [35]. No statistically significant differences were generally detected between genders in previous studies [18,36]. Recently, Ferrillo et al. reported a significant relation between female sex and premature midpalatal suture maturation [33]. However, the authors underlined the help of CBCT during clinical decision making in preadolescent males, as a fused suture could be found in circumpubertal ages.

Regarding the skeletal vertical growth pattern, in the current study, the hypodivergent and normodivergent subjects exhibited higher stages of midpalatal suture maturation.

The studies now available in the literature have mainly investigated the relation between suture development and vertical proportions [33,37]. Previous papers demonstrated that some anatomical structures, e.g., mandibular canal or muscle length, could vary according to the facial divergence [38]. The hyperdivergent subjects usually show a late facial growth associated with a longer mandible [39]. Oliveira et al. noticed a significant correlation between vertical facial pattern and palatal maturation [37]. The authors also observed stage B or C in hyperdivergent adults and, thus, stressed the use of CBCT in these patients during the diagnostic phase. On the contrary, in a recent paper, the vertical growth pattern was not suggested as a significant signal for midpalatal maturation staging [33].

Regarding the sagittal relationships, a uniform distribution of skeletal classes has been observed in relation to age and stages of palatal suture maturation. There appears to be a prevalence of stage C and D in all the skeletal classes, just because our study sample had a majority of patients in the middle age of the range (8–20 years). The incidence of suture maturation stages was the same across different skeletal classes. Therefore, according to the current study, no correlation was found between midpalatal suture maturation and skeletal class.

In the literature, no previous studies analysed the effect of anteroposterior skeletal pattern on suture development. However, some peculiarities related to sagittal growth pattern were described; for instance, a thinner palatal bone was detected in Class III compared to Class I subjects [40]. A correlation between skeletal class and palatal suture maturation could be useful in orthodontic diagnosis to understand which type of orthodontic approach to undertake. There were many comparative CBCT studies regarding the Class III cases that described the anatomy and different shape of the maxillary arch in Class III. However, these papers did not mention any correlations between maxillary bone growth and the stage of palatal suture maturation. Ahm et al. reported that the palatal morphology in skeletal Class III malocclusion differed from that in other sagittal relationships, as the mandibular development does not influence the maxillary growth, that remains underdeveloped. The researchers noted that in individuals with prognathism, as the width of the face increases, the palate tends to become more restricted, higher, and longer [41]. Similarly, Paoloni et al. used three dimensional images to investigate the palatal shape in Class III young patients. They founded that in those individuals, the increase of mandibular plane angle was associated with a restricted and deep palate [42]. Huang et al. reconstructed, three-dimensionally, the palate by CBCT scan. Class II patients with a retrusive mandible had a more reduced posterior portion of the palate than ones with a balanced profile; however, this research did not analyse the palatal shape of prognathic patients with different vertical proportions [43]. In another study, Huang et al. detected that, at the same divergence, skeletal Class III patients showed a flatter and more reduced posterior portion of the palate than skeletal Class I ones [44].

Based on the existing literature, the relation between suture maturation and the potential predictors involved in its development have yet to be fully understood, probably since the studies have differed in terms of their study sample selections, for example, including various age ranges with a limited number of participants. Moreover, including patients with a previous orthodontic treatment could distort the results, as orthodontic/orthopaedic expansion forces could affect the midpalatal suture development.

The current study was a CBCT analysis of the midpalatal suture. To reduce radiation exposure, we chose a low-dose CBCT protocol in our study. The low-dose CBCT provides an accurate 3D evaluation of the maxillary structure without the superimposition of the vomer and other surrounding anatomies. Moreover, the low-dose CBCT with an effective dose of 35 μSv allows a more in-depth diagnosis than other radiological methods used before the beginning of orthodontic therapy. Indeed, we requested, for our patients, CBCT scans prior to orthodontic treatment to add further diagnostic parameters in the presence of a skeletal malocclusion or impacted teeth.

Therefore, it is important to identify the potential indicators involved in midpalatal suture maturation in order to obtain a more precise diagnosis, and, consequently, a more predictable therapy result.

Our research revealed some limitations. Firstly, other biological variables, such as bone density, palate thickness, and fronto-maxillary, zygomatic-maxillary, zygomatic-frontal, and zygomatic-temporal sutures, may influence the resistance to maxillary transverse expansion and, consequently, midpalatal suture development [45,46]. Secondly, we limited our sample to the Caucasian race. Possible variations on palatal suture could be detected among different ethnic groups. Lastly, the number of participants in every age group was different; however, most subjects were 12- and 13-year-olds, i.e., the age groups in which there was a greater variability of midpalatal maturation.

Future research could be conducted prospectively to provide additional information concerning the relationship between various parameters and midpalatal suture development. Moreover, the transversal dentoalveolar expansion approaches could be further examined, especially in adult patients with a fused suture, who are generally candidates for a surgical technique.

The present study encompassed a sizeable and homogenous sample in a limited age range and investigated the influence of demographic and skeletal factors on the development and maturation of the midpalatal suture.

## 5. Conclusions

We conducted an exhaustive analysis concerning the midpalatal suture maturation in relation to age, sex, and vertical and sagittal growth patterns among 263 Italian individuals aged from 8 to 20. We found 48.29% of participants with unfused suture. The youngest and male patients as well as the hyperdivergent subjects showed lower maturation stages. Hence, the midpalatal suture maturation was related to age, sex, and divergence. Therefore, our analysis provides an aid for the clinicians in decision making. Clinically, the orthodontist can confidently choose a conventional expansion treatment for young, male, and hyperdivergent patients, as mainly skeletal effects would be achieved. An early maxillary expansion is essential to obtain a favourable growth of the mid and lower face and to avoid more complex therapies in adulthood.

## Figures and Tables

**Table 1:** Distribution of the study sample.

Age	Females + Males	Females	Males
8	13	5	8
9	14	6	8
10	13	8	5
11	29	16	13
12	41	23	18
13	31	19	12
14	21	12	9
15	17	12	5
16	13	4	9
17	20	9	11
18	12	9	3
19	18	9	9
20	21	12	9
8–20	263	144	119

**Table 2:** Linear regression models.

Variables	Age Coef.	s.e.	*p*-Value	Confidence Interval
Male	0.94 ***	(0.23)	0.000	0.49–1.38
Stage B	1.42 **	(0.55)	0.010	0.34–2.50
Stage C	3.67 ***	(0.46)	0.000	2.77–4.58
Stage D	7.21 ***	(0.44)	0.000	6.33–8.08
Stage E	10.16 ***	(0.51)	0.000	9.16–11.16
Class II	−0.37	(0.25)	0.140	−0.86–0.12
Class III	−0.18	(0.35)	0.608	−0.86–0.50
Hypodivergent	−1.61 ***	(0.36)	0.000	−2.33–−0.90
Normodivergent	−1.60 ***	(0.26)	0.000	−2.11–−1.10
Constant	9.44 ***	(0.49)	0.000	8.47–10.41
Observations	263			
F (9, 253)	81.72		0.000	
R-squared	0.74			

Standard errors in parentheses; *** *p* < 0.001, ** *p* < 0.01.

**Table 3:** Distribution of the midpalatal maturation stages by age and sex.

Age	Sex	Stage A	Stage B	Stage C	Stage D	Stage E
8	Total	11 (84.62%)	2 (15.38%)	0	0	0
	F	4 (80.0%)	1 (20.0%)			
	M	7 (87.50%)	1 (12.50%)			
9	Total	9 (64.29%)	5 (35.71%)	0	0	0
	F	3 (50.0%)	3 (50.0%)			
	M	6 (75.0%)	2 (25.0%)			
10	Total	0	10 (76.90%)	3 (23.10%)	0	0
	F		5 (62.50%)	3 (37.50%)		
	M		5 (100%)	0		
11	Total	0	6 (20.69%)	20 (68.97%)	3 (10.34%)	0
	F		2 (12.50%)	12 (75.0%)	2 (12.50%)	
	M		4 (30.77%)	8 (61.54%)	1 (7.69%)	
12	Total	0	1 (2.44%)	30 (73.17%)	9 (21.95%)	1 (2.44%)
	F		0	15 (65.22%)	7 (30.43%)	1 (4.35%)
	M		1 (5.56%)	15 (83.33%)	2 (11.11%)	0
13	Total	0	1 (3.22%)	15 (48.39%)	13 (41.94%)	2 (6.45%)
	F		0	8 (42.10%)	9 (47.37%)	2 (10.53%)
	M		1 (8.33%)	7 (58.34%)	4 (33.33%)	0
14	Total	0	0	8 (38.10%)	11 (52.38%)	2 (9.52%)
	F			3 (25.0%)	7 (58.33%)	2(16.67%)
	M			5 (55.56%)	4 (44.44%)	0
15	Total	0	0	3 (17.65%)	11 (64.70%)	3 (17.65%)
	F			2 (16.67%)	8 (66.66%)	2 (16.67%)
	M			1 (20.0%)	3 (60.0%)	1 (20.0%)
16	Total	0	0	2 (15.38%)	8 (61.54%)	3 (23.08%)
	F			0	2 (50.0%)	2 (50.0%)
	M			2 (22.22%)	6 (66.67)	1 (11.11%)
17	Total	0	0	0	14 (70.0%)	6 (30.0%)
	F				5 (55.56%)	4 (44.44%)
	M				9 (81.82%)	2 (18.18%)
18	Total	0	0	0	7 (58.33%)	5 (41.67%)
	F				4 (44.44%)	5 (55.56%)
	M				3 (100%)	0
19	Total	0	0	1 (5.56%)	8 (44.44%)	9 (50.0%)
	F			0	3 (33.33%)	6 (66.67%)
	M			1 (11.11%)	5 (55.56%)	3 (33.33%)
20	Total	0	0	0	8 (38.10%)	13 (61.90%)
	F				4 (33.33%)	8 (66.67%)
	M				4 (44.44%)	5 (55.56%)
8–20	Total	20 (7.60%)	25 (9.51%)	82 (31.18%)	92 (34.98%)	44 (16.73%)
	F	7 (4.86%)	11 (7.64%)	43 (29.86%)	51 (35.42%)	32 (22.22%)
	M	13 (10.92%)	14 (11.76%)	39 (32.77%)	41 (34.45%)	12 (10.10%)

F indicates female; M indicates male.

**Table 4:** Distribution of the study sample based on midpalatal stages and divergence.

Age (Years)	Vertical Growth Pattern	Stage A	Stage B	Stage C	Stage D	Stage E	Stages A–E
8	Hypodivergent	2 (15.39%)	1 (7.69%)	0	0	0	3 (23.08%)
		0 F	0 F				0 F
		2 M (25.0%)	1M (12.50%)				3M (37.50%)
	Normodivergent	8 (61.54%)	1 (7.69%)	0	0	0	9 (69.23%)
		4 F (80.0%)	1 F (20.0%)				5 F (100%)
		4 M (50.0%)	0 M				4 M (50.0%)
	Hyperdivergent	1 (7.69%)	0	0	0	0	1 (7.69%)
		0 F					0 F
		1 M (12.50%)					1 M (12.50%)
9	Hypodivergent	0	0	0	0	0	0
	Normodivergent	5 (35.71%)	5 (35.71%)	0	0	0	10 (71.42%)
		3 F (50.0%)	3 F (50.0%)				6 F (100%)
		2 M (25.0%)	2 M (25.0%)				4 M (25.0%)
	Hyperdivergent	4 (28.58%)	0	0	0	0	4 (28.58%)
		0 F					0 F
		4 M (50.0%)					4 M (50.0%)
10	Hypodivergent	0	1 (7.69%)	0	0	0	1 (7.69%)
			0 F				0 F
			1 M (20.0%)				1 M (20.0%)
	Normodivergent	0	2 (15.38%)	2 (15.38%)	0	0	4 (30.76%)
			1 F (12.50%)	2 F (25.0%)			3 F (37.50%)
			1 M (20.0%)	0 M			1 M (20.0%)
	Hyperdivergent	0	7 (53.86%)	1 (7.69%)	0	0	8 (61.55%)
			4 F (50.0%)	1 F (12.50%)			5 F (62.50%)
			3 M (60.0%)	0 M			3 M (60.0%)
11	Hypodivergent	0	0	4 (13.79%)	1 (3.45%)	0	5 (17.24%)
				3 F (18.75 %)	0 F		3 F (18.75%)
				1 M (7.69%)	1 M (7.69%)		2 M (15.38%)
	Normodivergent	0	2 (6.90%)	11 (37.93%)	2 (6.90%)	0	15 (51.73%)
			0 F	5 F (31.25%)	2 F (12.50%)		7 F (43.75%)
			2 M (15.39%)	6 M (46.14%)	0 M		8 M (61.53%)
	Hyperdivergent	0	4 (13.79%)	5 (17.24%)	0	0	9 (31.03%)
			2 F (12.50%)	4 F (25.0%)			6 F (37.50%)
			2 M (15.39%)	1 M (7.69%)			3 M (23.08%)
12	Hypodivergent	0	0	3 (7.32%)	2 (4.88%)	0	5 (12.20%)
				1 F (4.35%)	2 F (8.69%)		3 F (13.04%)
				2 M (11.11%)	0 M		2 M (11.11%)
	Normodivergent	0	0	8 (19.51%)	6 (14.63%)	1 (2.44%)	15 (36.58%)
				3 F (13.04%)	4 F (17.39%)	1 F (4.35%)	8 F (34.78%)
				5 M (27.78%)	2 M (11.11%)	0 M	7 M (38.89%)
	Hyperdivergent	0	1 (2.44%)	19 (46.34%)	1 (2.44%)	0	21 (51.22%)
			0 F	11 F (47.83%)	1 F (4.35%)		12 F (52.18%)
			1 M (5.56%)	8 M (44.44%)	0 M		9 M (50.0%)
13	Hypodivergent	0	0	0	3 (9.67%)	1 (3.23%)	4 (12.90%)
					2 F (10.53%)	1F (5.26%)	3 F (15.79%)
					1 M (8.33%)	0 M	1 M (8.33%)
	Normodivergent	0	0	1 (3.23%)	7 (22.58%)	1 (3.23%)	9 (29.04%)
				0 F	5 F (26.32%)	1 F (5.26%)	6 F (31.58%)
				1 M (8.33%)	2 M (16.68%)	0 M	3 M (25.01%)
	Hyperdivergent	0	1 (3.23%)	14 (45.16%)	3 (9.67%)	0	18 (58.06%)
			0 F	8 F (42.10%)	2 F (10.53%)		10 F (52.63%)
			1 M (8.33%)	6 M (50.0%)	1 M (8.33%)		8 M (66.66%)
14	Hypodivergent	0	0	0	0	2 (9.52%)	2 (9.52%)
						2 F (16.67%)	2 F (16.67%)
						0 M	0 M
	Normodivergent	0	0	4 (19.05%)	7 (33.33%)	0	11 (52.38%)
				2 F (16.67%)	3 F (25.0%)		5 F (41.67%)
				2 M (22.22%)	4 M (44.45%)		6 M (66.67%)
	Hyperdivergent	0	0	4 (19.05%)	4 (19.05%)	0	8 (38.10%)
				1 F (8.33%)	4 F (33.33%)		5 F (41.66%)
				3 M (33.33%)	0 M		3 M (33.33%)
15	Hypodivergent	0	0	0	1 (5.88%)	0	1(5.88%)
					1 F (8.33%)		1F (8.33%)
					0 M		0 M
	Normodivergent	0	0	2 (11.76%)	9 (52.94%)	3 (17.65%)	14 (82.35%)
				1 F (8.33%)	6 F (50.0%)	2 F (16.67%)	9 F (75.0%)
				1 M (20.0%)	3 M (60.0%)	1 M (20.0%)	5 M (100.0%)
	Hyperdivergent	0	0	1 (5.88%)	1 (5.88%)	0	2 (11.76%)
				1 F (8.33%)	1 F (8.33%)		2 F (16.67%)
				0 M	0 M		0 M
16	Hypodivergent	0	0	0	0	1 (7.69%)	1 (7.69%)
						1 F (25.0%)	1 F (25.0%)
						0 M	0 M
	Normodivergent	0	0	0	7 (53.84%)	2 (15.39%)	9 (69.23%)
					2 F (50.0%)	1 F (25.0%)	3 F (75.0%)
					5 M (55.56%)	1 M (11.11%)	6 M (66.67%)
	Hyperdivergent	0	0	2 (15.39%)	1(7.69%)	0	3 (23.08%)
				0 F	0 F		0 F
				2 M (22.22%)	1 M (11.11%)		3M (33.33%)
17	Hypodivergent	0	0	0	3 (15.0%)	6 (30.0%)	9 (45.0%)
					0 F	4 F (44.45%)	4F (44.45%)
					3 M (27.27%)	2 M (18.19%)	5 M (45.46%)
	Normodivergent	0	0	0	6 (30.0%)	0	6 (30.0%)
					3 F (33.33%)		3 F (33.33%)
					3 M (27.27%)		3 M (27.27%)
	Hyperdivergent	0	0	0	5 (25.0%)	0	5 (25.0%)
					2 F (22.22%)		2 F (22.22%)
					3 M (27.27%)		3M (27.27%)
18	Hypodivergent	0	0	0	0	2 (16.67%)	2 (16.67%)
						2 F (22.22%)	2F (22.22%)
						0 M	0 M
	Normodivergent	0	0	0	0	1 (8.33%)	1 (8.33%)
						1 F (11.12%)	1 F (11.12%)
						0 M	0 M
	Hyperdivergent	0	0	0	7 (58.33%)	2 (16.67%)	9 (75.0%)
					4 F (44.44%)	2 F (22.22%)	6 F (66.66%)
					3 M (100%)	0 M	3 M (100%)
19	Hypodivergent	0	0	0	1 (5.56%)	4 (22.22%)	5 (27.78%)
					0 F	3 F (33.34%)	3 F (33.34%)
					1 M (11.11%)	1 M (11.11%)	2 M (22.22%)
	Normodivergent	0	0	0	0	3 (16.66%)	3 (16.66%)
						2 F (22.22%)	2 F (22.22%)
						1 M (11.11%)	1 M (11.11%)
	Hyperdivergent	0	0	1 (5.56%)	7 (38.89%)	2 (11.11%)	10 (55.56%)
				0 F	3 F (33.33%)	1 F (11.11%)	4 F (44.44%)
				1 M (11.11%)	4 M (44.45%)	1 M (11.11%)	6 M (66.67%)
20	Hypodivergent	0	0	0	0	7 (33.33%)	7 (33.33%)
						5 F (41.67%)	5 F (41.67%)
						2 M (22.22%)	2 M (22.22%)
	Normodivergent	0	0	0	1 (4.77%)	6 (28.57%)	7 (33.34%)
					0 F	3 F (25.0%)	3 F (25.0%)
					1 M (11,12%)	3 M (33.33%)	4 M (44.45%)
	Hyperdivergent	0	0	0	7 (33.33%)	0	7 (33.33%)
					4 F (33.33%)		4 F (33.33%)
					3 M (33.33%)		3 M (33.33%)
Total	Hypodivergent	2 (0.76%)	2 (0.76%)	7 (2.66%)	11 (4.18%)	23 (8.75%)	45 (17.11%)
		0 F	0 F	4 F (2.78%)	5 F (3.47%)	18 F (12.50%)	27 F (18.75%)
		2 M (1.68%)	2 M (1.68%)	3 M (2.52%)	6 M (5.04%)	5 M (4.20%)	18 M (15.12%)
	Normodivergent	13 (4.94%)	10 (3.80%)	28 (10.65%)	45 (17.11%)	17 (6.47%)	113 (42.97%)
		7 F (4.86%)	5 F (3.47%)	13 F (9.03%)	25 F (17.36%)	11 F (7.64%)	61 F (42.36%)
		6 M (5.04%)	5 M (4.20%)	15 M (12.61%)	20 M (16.81%)	6 M (5.04%)	52 M (43.70%)
	Hyperdivergent	5 (1.90%)	13 (4.94%)	47 (17.87%)	36 (13.69%)	4 (1.52%)	105 (39.92%)
		0 F	6 F (4.17%)	26 F (18.06%)	21 F (14.58%)	3 F (2.08%)	56F (38.89%)
		5 M (4.20%)	7 M (5.88%)	21 M (17.65%)	15 M (12.61%)	1 M (0.84%)	49 M (41.18%)

F indicates female; M indicates male.

**Table 5:** Distribution of the study sample based on midpalatal stages and sagittal pattern.

Age (Years)	Sagittal Growth Pattern	Stage A	Stage B	Stage C	Stage D	Stage E	Stages A–E
8	Class I	3 (23.08%)	2 (15.38%)	0	0	0	5 (38.46%)
		1 F (20%)	1 F (20%)				2 F (40%)
		2 M (25%)	1 M (12.5%)				3 M (37.5%)
	Class II	6 (46.16%)	0	0	0	0	6 (46.16%)
		2 F (40%)					2 F (40%)
		4 M (50%)					4 M (50%)
	Class III	2 (15.38%)	0	0	0	0	2 (15.38%)
		1 F (20%)					1 F (20%)
		1 M (12.5%)					1 M (12.5%)
9	Class I	3 (21.43%)	1 (7.14%)	0	0	0	4 (28.57%)
		2 F (33.33%)	1 F (16.67%)				3 F (50%)
		1 M (12.5%)	0 M (0%)				1 M (12.5%)
	Class II	3 (21.43%)	4 (28.57%)	0	0	0	7 (50%)
		1 F (16.67%)	2 F (33.33%)				3 F (50%)
		2 M (25%)	2 M (25%)				4 M (50%)
	Class III	3 (21.43%)	0	0	0	0	3 (21.43%)
		0 F (0%)					0 F (0%)
		3 M (37.5%)					3 M (37.5%)
10	Class I	0	2 (15.39%)	1 (7.69%)	0	0	3 (23.08%)
			1 F (12.5%)	1 F (12.5%)			2 F (25.0%)
			1 M (20%)	0 M (0%)			1 M (20%)
	Class II	0	8 (61.54%)	2 (15.38%)	0	0	10 (76.92%)
			4 F (50%)	2 F (25%)			6 F (75%)
			4 M (80%)	0 M (0%)			4 M (80%)
	Class III	0	0	0	0	0	0
11	Class I	0	0	7 (24.14%)	0	0	7 (24.14%)
				5 F (31.25%)			5 F (31.25%)
				2 M (15.38%)			2 M (15.38%)
	Class II	0	5 (17.24%)	10 (34.48%)	3 (10.35%)	0	18 (62.07%)
			1 F (6.25%)	5 F (31.25%)	2 F (12.5%)		8 F (50%)
			4 M (30.77%)	5 M (38.47%)	1 M (7.69%)		10 M (76.93%)
	Class III	0	1 (3.45%)	3 (10.34%)	0	0	4 (13.79%)
			1 F (6.25%)	2 F (12.5%)			3 F (18.75%)
			0 M (0%)	1 M (7.69%)			1 M (7.69%)
12	Class I	0	1 (2.44%)	13 (31.7%)	2 (4.88%)	0	16 (39.02%)
			0 F (0%)	7 F (30.43%)	1 F (4.35%)		8 F (34.78%)
			1 M (5.56%)	6 M (33.33%)	1 M (5.55%)		8 (44.44%)
	Class II	0	0	16 (39.02%)	4 (9.76%)	1 (2.44%)	21 (51.22%)
				7 F (30.44%)	3 F (13.04%)	1 F (4.35%)	11 F (47.83%)
				9M (50%)	1 M (5.56%)	0 M (0%)	10 M (55.56%)
	Class III	0	0	1 (2.44%)	3 (7.32%)	0	4 (9.76%)
				1 F (4.35%)	3 F (13.04%)		4 F (17.39%)
				0 M (0%)	0 M (0%)		0 M (0%)
13	Class I	0	0	5 (16.13%)	3 (9.68%)	0	8 (25.81%)
				4 F (21.05%)	3 F (15.79%)		7 F (36.84%)
				1 M (8.33%)	0 M (0%)		1 M (8.33%)
	Class II	0	1 (3.23%)	8 (25.81%)	8 (25.81%)	2 (6.45%)	19 (61.29%)
			0 F (0%)	3 F (15.79%)	6 F (31.58%)	2 F (10.53%)	11 F (57.9%)
			1 M (8.33%)	5 M (41.67%)	2 M (16.67%)	0 M (0%)	8 (66.67%)
	Class III	0	0	2 (6.45%)	2 (6.45%)	0	4 (12.9%)
				1 F (5.26%)	0 F (0%)		1 F (5.26%)
				1 M (8.33%)	2 M (16.67%)		3 M (25%)
14	Class I	0	0	2 (9.52%)	4 (19.05%)	0	6 (28.57%)
				0 F (0%)	4 F (33.33%)		4 F (33.33%)
				2 M (22.22%)	0 M (0%)		2 M (22.22%)
	Class II	0	0	5 (23.81%)	5 (23.81%)	2 (9.52%)	12 (57.14%)
				3 F (25%)	1 F (8.33%)	2 F (16.67%)	6 F (50%)
				2 M (22.22%)	4 M (44.45%)	0 M (0%)	6 M (66.67%)
	Class III	0	0	1 (4.76%)	2 (9.53%)	0	3 (14.29%)
				0 F (0%)	2 F (16.67%)		2 F (16.67%)
				1 M (11.11%)	0 M (0%)		1 M (11.11%)
15	Class I	0	0	1 (5.88%)	4 (23.53%)	1 (5.89%)	6 (35.3%)
				1 F (8.33%)	3 F (25%)	0 F (0%)	4 F (33.33%)
				0 M (0%)	1 M (20%)	1 M (20%)	2 M (40%)
	Class II	0	0	2 (11.76%)	4 (23.53%)	2 (11.76%)	8 (47.05%)
				1 F (8.33%)	2 F (16.67%)	2 F (16.67%)	5 F (41.67%)
				1 M (20%)	2 M (40%)	0 M (0%)	3 M (60%)
	Class III	0	0	0	3 (17.65%)	0	3 (17.65%)
					3 F (25%)		3 F (25%)
					0 M (0%)		0 M (0%)
16	Class I	0	0	1 (7.69%)	5 (38.47%)	1 (7.69%)	7 (53.85%)
				0 F (0%)	0 F (0%)	0 F (0%)	0 F (0%)
				1 M (11.11%)	5 M (55.56%)	1 M (11.11%)	7 M (77.78%)
	Class II	0	0	0	2 (15.38%)	2 (15.39%)	4 (30.77%)
					1 F (25%)	2 F (50%)	3 F (75%)
					1 M (11.11%)	0 M (0%)	1 M (11.11%)
	Class III	0	0	1 (7.69%)	1 (7.69%)	0	2 (15.38%)
				0 F (0%)	1 F (25%)		1 F (25%)
				1 M (11.11%)	0 M (0%)		1 M (11.11%)
17	Class I	0	0	0	6 (30%)	0	6 (30%)
					4 F (44.45%)		4 F (44.45%)
					2 M (18.18%)		2 M (18.18%)
	Class II	0	0	0	5 (25%)	5 (25%)	10 (50%)
					1 F (11.11%)	3 F (33.33%)	4 F (44.44%)
					4 M (36.37%)	2 M (18.18%)	6 M (54.55%)
	Class III	0	0	0	3 (15%)	1 (5%)	4 (20%)
					0 F (0%)	1 F (11.11%)	1 F (11.11%)
					3 M (27.27%)	0 M (0%)	3 M (27.27%)
18	Class I	0	0	0	2 (16.67%)	2 (16.67%)	4 (33.34%)
					1 F (11.11%)	2 F (22.22%)	3 F (33.33%)
					1 M (33.33%)	0 M (0%)	1 M (33.33%)
	Class II	0	0	0	3 (25%)	1 (8.33%)	4 (33.33%)
					2 F (22.23%)	1 F (11.11%)	3 F (33.34%)
					1 M (33.33%)	0 M (0%)	1 M (33.33%)
	Class III	0	0	0	2 (16.67%)	2 (16.67%)	4 (33.33%)
					1 F (11.11%)	2 F (22.22%)	3 F (33.33%)
					1 M (33.34%)	0 M (0%)	1 M (33.34%)
19	Class I	0	0	1 (5.56%)	1 (5.55%)	4 (22.22%)	6 (33.33%)
				0 F (0%)	0 F (0%)	3 F (33.33%)	3 F (33.33%)
				1 M (11.11%)	1 M (11.11%)	1 M (11.11%)	3 M (33.33%)
	Class II	0	0	0	4 (22.22%)	3 (16.67%)	7 (38.89%)
					1 F (11.11%)	3 F (33.34%)	4 F (44.45%)
					3 M (33.33%)	0 M (0%)	3 M (33.33%)
	Class III	0	0	0	3 (16.67%)	2 (11.11%)	5 (27.78%)
					2 F (22.22%)	0 F (0%)	2 F (22.22%)
					1 M (11.11%)	2 M (22.23%)	3 M (33.34%)
20	Class I	0	0	0	4 (19.05%)	4 (19.05%)	8 (38.1%)
					2 F (16.67%)	1 F (8.33%)	3 F (25%)
					2 M (22.22%)	3 M (33.34%)	5 M (55.56%)
	Class II	0	0	0	2 (9.52%)	8 (38.1%)	10 (47.62%)
					2 F (16.67%)	6 F (50%)	8 F (66.67%)
					0 M (0%)	2 M (22.22%)	2 M (22.22%)
	Class III	0	0	0	2 (9.52%)	1 (4.76%)	3 (14.28%)
					0 F (0%)	1 F (8.33%)	1 F (8.33%)
					2 M (22.22%)	0 M (0%)	2 M (22.22%)
Total	Class I	6 (2.28%)	6 (2.28%)	31 (11.79%)	31 (11.79%)	12 (4.56%)	86 (32.7%)
		3 F (2.08%)	3 F (2.08%)	18 F (12.5%)	18 F (12.5%)	6 F (4.17%)	48 F (33.33%)
		3 M (2.52%)	3 M (2.52%)	13 M (10.92%)	13 M (10.92%)	6 M (5.05%)	38 M (31.93%)
	Class II	9 (3.42%)	18 (6.84%)	43 (16.35%)	40 (15.21%)	26 (9.89%)	136 (51.71%)
		3 F (2.08%)	7 F (4.86%)	21 F (14.58%)	21 F (14.58%)	22 F (15.29%)	74 F (51.39%)
		6 M (5.04%)	11 M (9.24%)	22 M (18.49%)	19 M (15.97%)	4 M (3.36%)	62 M (52.1%)
	Class III	5 (1.9%)	1 (0.38%)	8 (3.04%)	21 (7.98%)	6 (2.29%)	41 (15.59%)
		1 F (0.69%)	1 F (0.7%)	4 F (2.78%)	12 F (8.33%)	4 F (2.78%)	22 F (15.28%)
		4 M (3.36%)	0 M (0%)	4 M (3.36%)	9 M (7.56%)	2 M (1.69%)	19 M (15.97%)

F indicates female; M indicates male.

## Data Availability

The original contributions presented in the study are included in the article, further inquiries can be directed to the corresponding author.
